# Bibliometric analysis of research themes and trends in childhood autism spectrum disorders from 2012 to 2021

**DOI:** 10.3389/fpubh.2022.925475

**Published:** 2022-08-31

**Authors:** Junqiang Zhao, Yi Lu, Xingyang Wu, Fujun Zhou, Fangqin Fei, Xiaoyan Wu, Xiufang Ding, Minli Wang

**Affiliations:** ^1^Department of Nursing, Xinxiang Medical University, Xinxiang, China; ^2^Department of Medical Engineering, Xinxiang Medical University, Xinxiang, China; ^3^Department of Children Rehabilitation, The First Affiliated Hospital of Xinxiang Medical University, Xinxiang, China; ^4^Department of Nursing, The First Affiliated Hospital of Huzhou University, Huzhou, China; ^5^Department of Nursing, Huzhou Maternal and Child Health Care Hospital, Huzhou, China

**Keywords:** autism spectrum disorders, children, bibliometrics, CiteSpace, VOSviewer

## Abstract

**Background:**

Autism spectrum disorders (ASD) are heterogeneous neurodevelopmental conditions that affect people worldwide. Early diagnosis and clinical support help achieve good outcomes. However, medical system structure and restricted resource availability create challenges that increase the risk of poor outcomes. Understanding the research progress of childhood ASD in recent years, based on clinical literature reports, can give relevant researchers and rehabilitation therapists more resonable research guides.

**Objective:**

This bibliometric study aimed to summarize themes and trends in research on childhood ASD and to suggest directions for future enquiry.

**Methods:**

Citations were downloaded from the Web of Science Core Collection database on childhood ASD published from 1 January 2012, to 31 December 2021. The retrieved information was analyzed using CiteSpace.5.8. R3, and VOS viewer.

**Results:**

A total of 7,611 papers were published across 103 areas. The United States was the leading source of publications. The clusters that have continued into 2020 include coronavirus disease 2019, gut microbiota, and physical activity, which represent key research topics. Keywords with frequency spikes during 2018–2021 were “disabilities monitoring network,” “United States,” and “caregiver.”

**Conclusions:**

The Autism and Developmental Disabilities Monitoring Network in the United States can be used as a reference for relevant workers worldwide. An intelligent medical assistant system is being developed. Further studies are required to elucidate challenges associated with caring for a child with ASD.

## Introduction

Autism spectrum disorders (ASD) are heterogeneous neurodevelopmental conditions, characterized by early-onset difficulties in communication and restricted, repetitive behavior and interests (1). In 2020, the World Health Organization reported that 1 in 160 children worldwide presents with ASD (2). The United States National Center for Health Statistics reported in 2016 that the prevalence of ASD among children aged 3 to 17 years is 1 in 45 and that it is increasing, which is a cause for concern. ASD is a lifelong neurodevelopmental disorder with a profound impact on intellectual and general abilities and psychological functioning (3, 4). ASD was first described based on the characteristics of 11 children observed by child psychiatrist Kanner in 1943 (5). The definition of ASD continues to evolve (1, 6–8). The understanding of ASD is also evolving, and the number of related studies is increasing. Examining what currently constitutes research frontiers may help set directions for future research (9). This study aimed to examine the current evidence on childhood ASD, including research trends and leading topics, published over the past decade, and to propose directions for future research. These findings may be used as a reference for epidemiologists, pediatricians, rehabilitation therapists, and caregivers.

This study aimed to cover the following aspects. We analyzed Science Citation Index for childhood ASD studies using bibliometric methods. Countries, regions, institutions, and periodicals of study origin, study categories, keywords, and references per study were included in the dataset. Visual representation of findings was provided. Research influence of countries, regions, institutions, and journals was examined, and leading research topics were reported. We went over the hot spot trend in greater depth.

## Materials and methods

**Figure 1:**
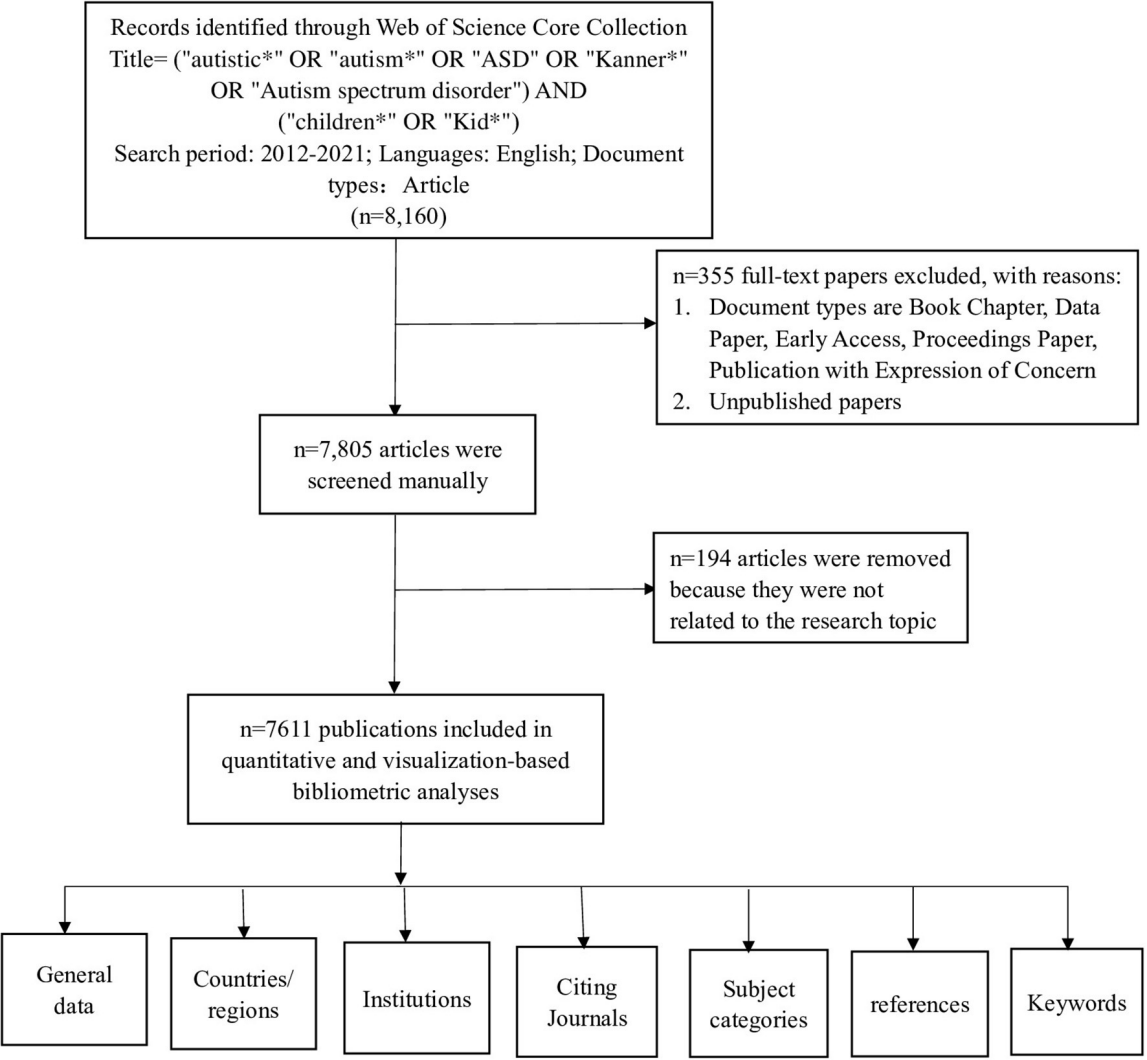
Study flow diagram shows eligibility criteria and bibliometric analysis protocol for childhood Autism Spectrum Disorders (ASD).

On 24 March 2022, all citations published between 1 January 2012, and 31 December 2021, were retrieved from the Web of Science Core Collection (WoSCC). The search strategy was set as TI = (“autistic^*^” OR “autism^*^” OR “ASD” OR “Kanner^*^” OR “Autism spectrum disorder”) AND TI = (children OR Kid^*^), the document type was “ARTICLE,” and the timeframe was from 1 January 2012 to 31 December 2021. A total of 8,160 papers were retrieved. After manual screening, 7,611 articles were included, and data on the following variables were extracted: title, publication year, country or region, institution, journal, references, and keywords (Figure 1).

### Statistical analysis

Citation features were analyzed using CiteSpace.5.8. R3 and VOSviewer. The H-index was used to estimate the importance or impact of citations obtained from WoSCC.

## Results

### Annual distribution of publications

**Figure 2:**
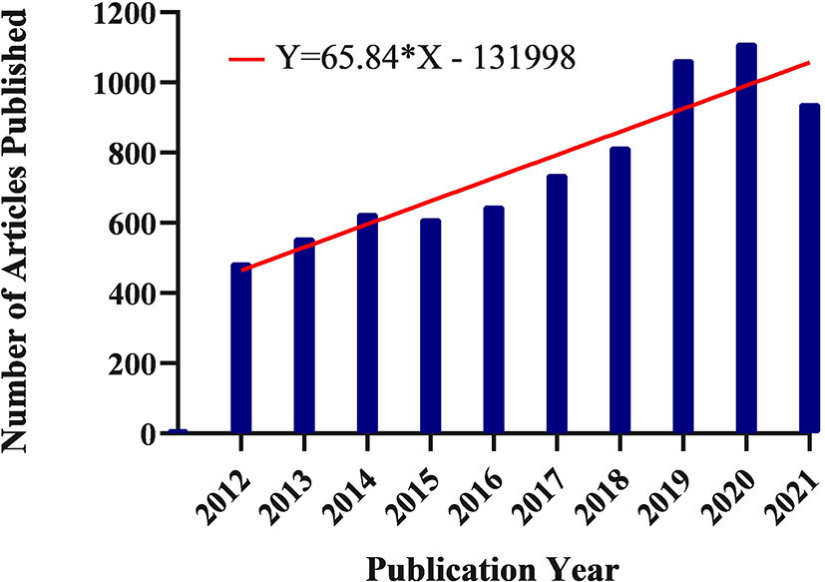
Number of papers published on childhood ASD from 2012 to 2021.

Both the retrieval result analyzer of Web of Science database and the duplicate removal function of CiteSpace software can obtain the number of documents issued each year. Over the past 10 years, the number of studies on childhood ASD has increased (Figure 2). The number of publications in this area increased from 2011 to 2018. More than 1,000 research papers were published in this area from 2018 to 2020. Finally, from 2020 to 2021, the number of documents decreased slightly.

### Countries or regions

**Figure 3:**
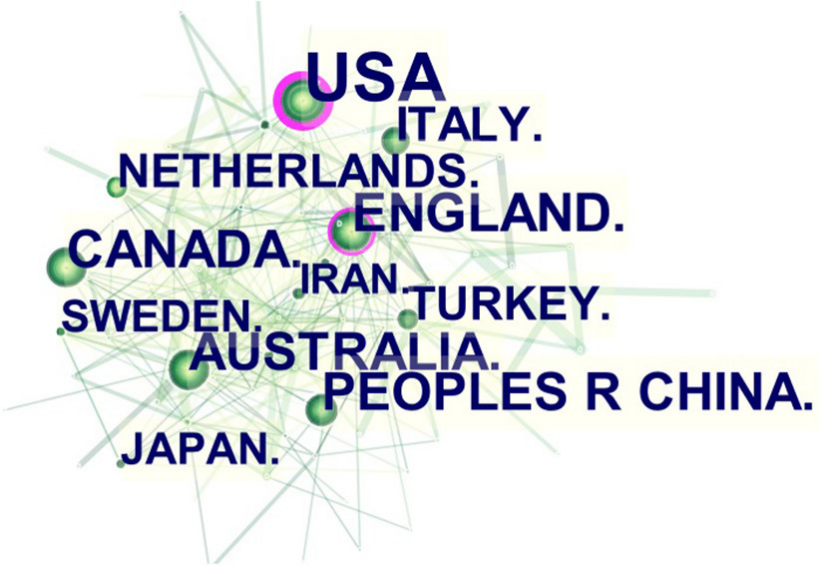
Collaborations of countries or regions reporting studies on childhood ASD from 2012 to 2021.

Using the country cooperation analysis function of CiteSpace, we can get the number of countries that have published articles and the influence of articles from a country. A total of 7,611 papers were published in 103 countries or regions. Collaborations among countries/regions are shown in Figure 3. The number of publications corresponds to the size of tags or nodes. Centrality can be ascertained from the purple ring area. Regions with the greatest number of citations included the United States (*n* = 3,655), Britain (*n* = 653), Australia (*n* = 553), China (*n* = 478), and Canada (*n* = 472). Among them, the United States (0.40) and Britain (0.17) have purple rings, representing articles from these regions have been cited more frequently than articles from other countries (Table 1). The H-index represents citation influence. The three countries with the highest h-index are the United States (102), England (58) and Canada (50).

**Table 1:** Top 10 countries or regions with publications on childhood ASD from 2012 to 2021.

**Rank**	**Country/region**	**Count**	**Centrality**	**H-index**
1	United States	3,655	0.40	102
2	England	653	0.17	58
3	Australia	553	0.09	45
4	Peoples Republic of China	478	0.02	34
5	Canada	472	0.02	50
6	Italy	319	0.04	36
7	Turkey	222	0.04	20
8	Netherlands	206	0.03	39
9	Japan	172	0.00	22
10	Iran	165	0.00	22

### Institutions

**Figure 4:**
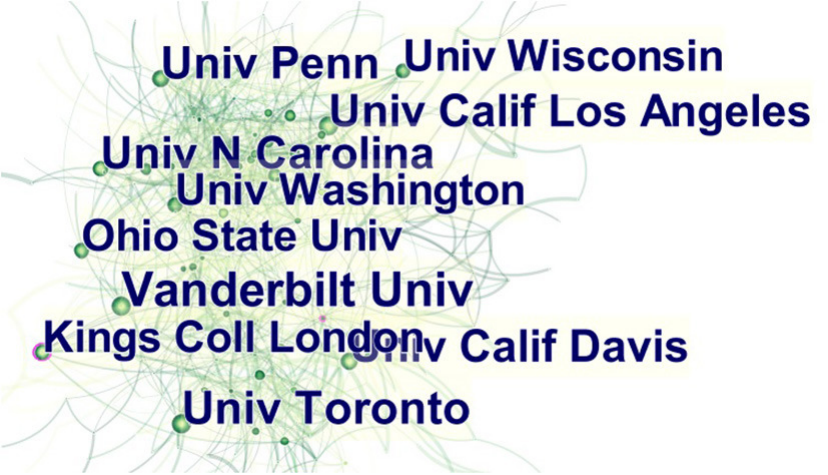
Collaborations of institutions reporting studies on childhood ASD from 2012 to 2021.

The cooperation between institutions and the number of published articles of each institution can be achieved through CiteSpace software. Table 2 lists the top ten institutions with the largest number of documents, and their partnerships are shown in Figure 4. Eight of them are located in the United States, one in Canada, and one in the United Kingdom. The total link strength shows the influence of each institution.

**Table 2:** Top 10 institutions with publications on childhood ASD from 2012 to 2021.

**Rank**	**Institutions**	**Country**	**Count**	**Total link strength**
1	Vanderbilt University	United States	166	4,409
2	University of Toronto	Canada	139	4,198
3	University of Pennsylvania	United States	124	3,839
4	University of California-Los Angeles	United States	122	3,641
5	University of California-Davis	United States	121	3,373
6	University of North Carolina	United States	118	3,321
7	University of Washington	United States	112	3,290
8	Ohio State University	United States	104	3,201
9	University of Wisconsin	United States	104	3,148
10	King's College London	England	102	2,269

### Journals and research category

**Figure 5:**
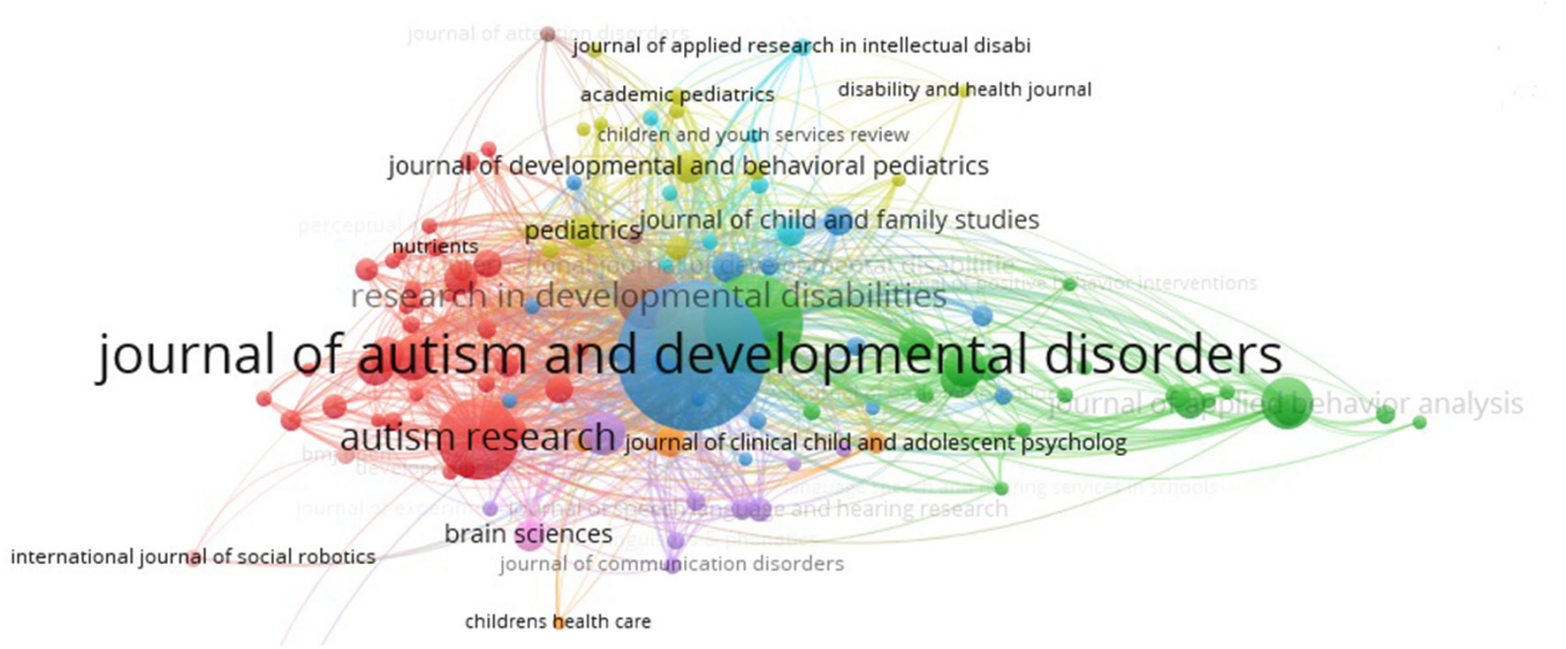
The cooperation of citiation journals that contributed to publications on Childhood ASD from 2012 to 2021.

Using VOSviewer to analyze the citation source journals and co cited journals. The documents in the highest citing journals represent leading research topics. The research field of highly cited journals are research basic knowledge. Tables 3, 4 show the top ten citing journals and cited journals, respectively. The leading research fields included psychology, special education, psychiatry, rehabilitation medicine, behavioral science, rehabilitation medicine, genetics, and neuroscience. Areas cited at increased frequencies included psychology, psychiatry, special education, rehabilitation medicine pediatrics, and behavioral science. The Journal of Autism and Developmental Disorders emerged as the most influential journal in this field (Figure 5).

**Table 3:** The top 10 citing journals of publications on Childhood from ASD 2012 to 2021.

**Rank**	**Citing journals**	**Research fields**	**Count**	**Journal impact factor in 2020**
1	Journal of Autism and Developmental Disorders	Psychology: development	1,070	4.291
2	Research in Autism Spectrum Disorders	Special education/psychology: development/Psychiatry/rehabilitation medicine	471	2.881
3	Autism	Psychology: development	348	5.689
4	Autism research	Behavioral science/psychology	319	5.216
5	Research in Developmental Disabilities	Special education/rehabilitation medicine	197	3.23
6	Journal of Applied Behavior Analysis	Psychology	135	3.695
7	Frontiers in Psychology	Psychology	93	2.988
8	Plos One	Comprehensive journal	88	3.24
9	Journal of Developmental and Physical Disabilities	Special education/psychology: development/rehabilitation medicine	86	1.71
10	Molecular Autism	Genetics/neuroscience	66	7.509

**Table 4:** Journal titles of the top 10 cited publications on childhood ASD from 2012 to 2021.

**Rank**	**Cited journals**	**Research field**	**Count**	**Journal impact factor in 2020**
1	Journal of Autism and Developmental Disorders	Psychology: development	6,342	4.291
2	Autism	Psychology: development	3,733	5.689
3	Journal of Child Psychology and Psychiatry	Psychology/psychiatry	3,261	8.982
4	Research in Autism Spectrum Disorders	Special education/psychology: development/psychiatry/rehabilitation medicine	3,233	2.881
5	Pediatrics	Medicine/pediatrics	2,378	7.125
6	Journal of the American Academy of Child and Adolescent Psychiatry	Pediatrics/psychiatry	2,334	8.829
7	Research in Developmental Disabilities	Special education/rehabilitation medicine	2,312	3.23
8	Autism Research	Behavioral science/psychology	2,224	5.216
9	PLoS One	Comprehensive journal	1,572	3.24
10	Child Development	Psychology: education	1,295	5.899

### Keywords

**Figure 6:**
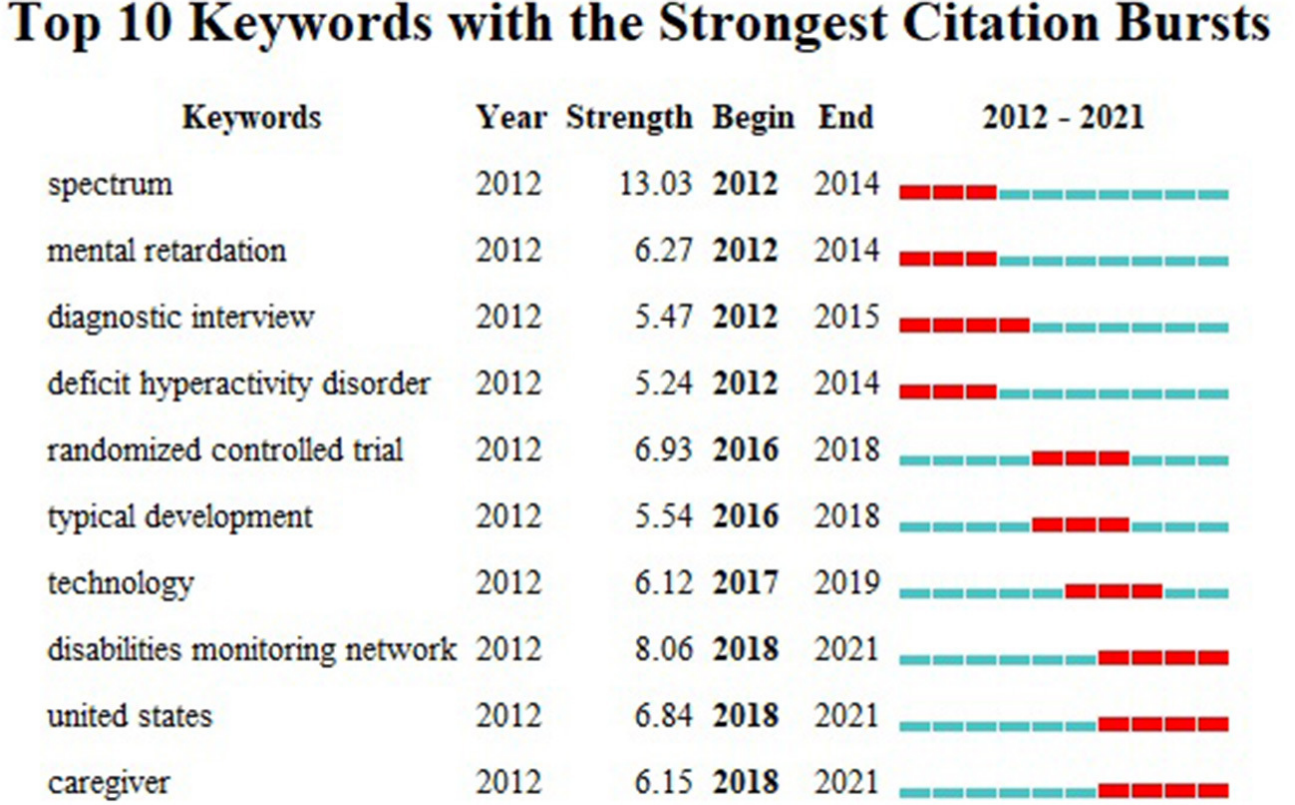
Keywords with the strongest citation bursts of publications on childhood ASD from 2012 to 2021.

The default setting of CiteSpace is changed to the following mode: “Year Per Slice” = 2, “Top N%” = 30.0%, and “Minimum Duration” = 2. Figure 6 summarizes the leading keywords, including the year of emergence. The red square in Figure 6 represents emerging keywords for the investigated timeline. The most frequently used keywords were “spectrum” (2012–2014), “deficit hyperactivity disorder” (2012–2014), “diagnostic interview” (2012–2015), “randomized controlled trial” (2016–2018), “typical development” (2016–2018), “technology” (2017–2019), “disabilities monitoring network” (2018–2021), “United States” (2018–2021), and “caregiver” (2018–2021).

### References

**Figure 7:**
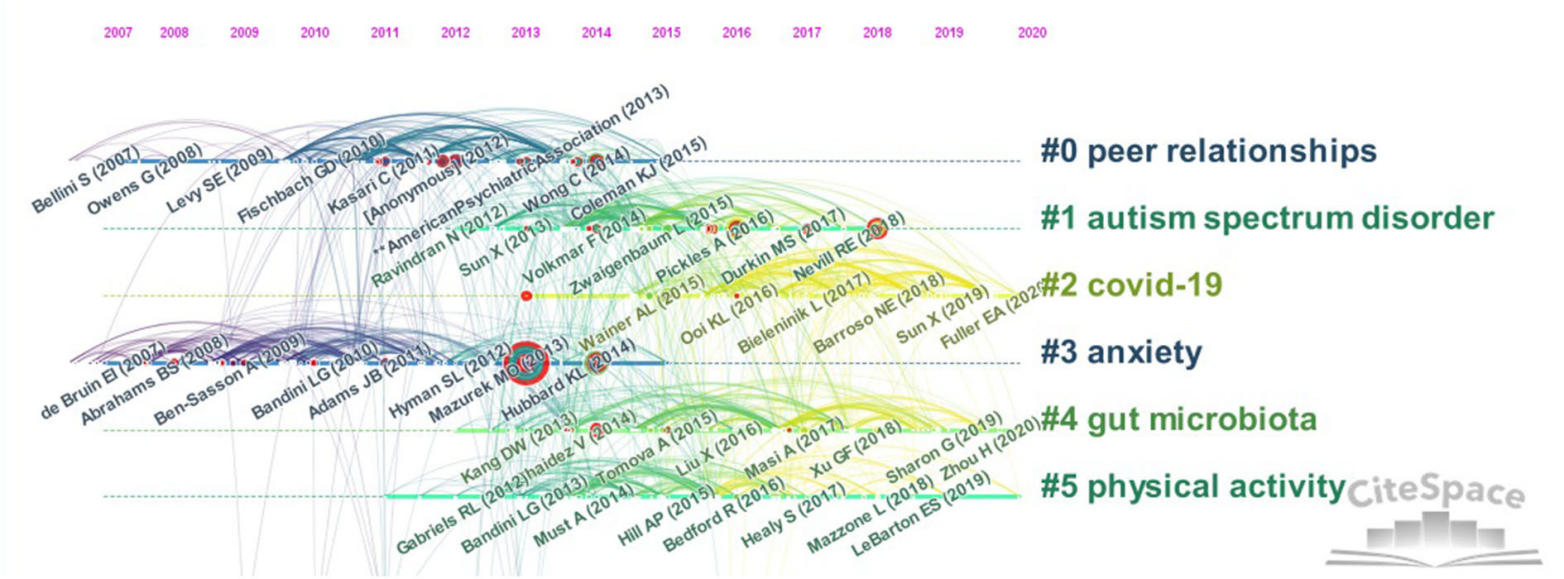
Timeline of publication co-citations on childhood ASD from 2012 to 2021.

Using the default setting of CiteSpace to cluster the co cited documents and choose label clusters with indexing terms. The clustering labels of co-cited articles depend on citing and cited journals. The cited studies constitute knowledge base of research. The frequency of reference use represents its influence. Co-citations reveal research themes and development background. Table 5 lists the top 10 cited studies, which include research of diagnostic criteria, intervention methods, epidemiological findings, and ASD characteristics. Two of these studies examined ASD within families. The clustering labels of cited documents are obtained from the citing documents. Figure 7 presents clusters that continued into 2020, including #2 COVID-19, #4 gut microbiota, and #5 physical activity.

**Table 5:** Top 10 most cited publications on childhood ASD from 2012 to 2021.

**Rank**	**Title of cited document**	**DOI**	**Count**	**Interpretation of findings**
1	Diagnostic and statistical manual of mental disorders, fifth edition, DSM V (10)	doi: 10.1176/appi.books.9780890425596	928	Provides diagnostic criteria of ASD
2	Prevalence of autism spectrum disorder among children aged 8 years- autism and developmental disabilities monitoring network, 11 sites, United States, 2014 (11)	doi: 10.15585/mmwr.ss6706a1	298	Provides the latest ASD prevalence estimates provided by the Autism and Developmental Disabilities Monitoring (ADDM) network according to DSMIV-TR and DSM-5 standards and asserts the need to monitor prevalence trends in ASD and to improve early diagnosis
3	The impact of parenting stress: a meta-analysis of studies comparing the experience of parenting stress in parents of children with and without autism spectrum disorder (12)	doi: 10.1007/s10803-012-1604-y	107	Reports that family stress associated with raising ASD children is higher than that associated with raising children with other types of disabilities or those with typical development
4	Autism (9)	doi: 10.1016/S0140-6736(13)61539-1	92	Provides ASD definition, epidemiology, prognosis, outcomes, early symptoms and screening characteristics, clinical evaluation criteria, cognitive and neuroscience findings, and neurobiological, genetic, and intervention characteristics
5	Randomized, controlled trial of an intervention for toddlers with autism: the early start denver model (13)	doi: 10.1542/peds.2009-0958	86	Presents evidence that comprehensive development behavior intervention for children with ASD helps improve cognitive and adaptive behavior and reduce ASD severity
6	Global prevalence of autism and other pervasive developmental disorders (14)	doi: 10.1002/aur.239	78	Systematically reviews global epidemiological findings on ASD and pervasive developmental disorders
7	Evidence-based practices for children, youth, and young adults with autism spectrum disorder: a comprehensive review (15)	doi: 10.1007/s10803-014-2351-z	77	Presents evidence-based and targeted interventions for children and adolescents with autism spectrum disorders
8	Naturalistic developmental behavioral interventions: empirically validated treatments for autism spectrum disorder (16)	doi: 10.1007/s10803-015-2407-8	75	Summarized the theoretical and empirical evidence for the naturalistic developmental behavior intervention and suggests directions for further research
9	What is the male-to-female ratio in autism spectrum disorder? A systematic review and meta-analysis (17)	doi: 10.1016/j.jaac.2017.03.013	71	Presents evidence that girls who meet the ASD criteria are at a high risk of not receiving a clinical diagnosis
10	Prevalence and characteristics of autism spectrum disorder among 4-year-old children in the autism and developmental disabilities monitoring network (18)	doi: 10.1097/DBP.0000000000000235	67	Provides evidence relevant to early identification of children with ASD, suggesting that assessment age should be reduced in in communities participating in autism and developmental disorder monitoring

## Discussion

### Principal results

The number of studies on childhood ASD has increased likely due to a combination of educational and medical system changes, which increased disease awareness. The number of published studies decreased from 2020 to 2021 likely due to restrictions associated with the coronavirus disease 2019 pandemic. The United States is the leading source of studies, suggesting policies and regulations in this country are more responsive to the needs of autism patients than those in other countries. The most influential journal in this field is the Journal of Autism and Developmental Disorders. This field includes disciplines such as psychology, psychiatry, special education, rehabilitation, medicine, pediatrics, and behavioral science. Meanwhile, keyword co-occurrence and reference cluster analysis revealed changes in research focus over time. From the emerging keywords from 2012 to 2015, the diagnosis of ASD and its comorbidity were the research hotspots of that year. In 2016 and 2017, we tend to establish randomized controlled trials within ASD groups and the differences between ASD and typical development groups. Based on previous research findings, researchers began looking for more appropriate technologies to establish disability monitoring networks and strengthen attention to ASD caregivers from 2017 to 2021.

### Research focus

#### Disability monitoring networks

Centers for Disease Control and Prevention (CDC) began monitoring the prevalence of ASD in 1996. The Children's Health Act of 2000 authorized CDC to establish the Autism and Developmental Disabilities Monitoring (ADDM) Network, which aims to assess ASD prevalence and diagnosis timing in children aged 4 and 8 years (19). The ADDM Network has established ASD and developmental disorder monitoring sites across the United States, starting with six sites in 2000 to 14 sites in 2002, and then to 8 sites in 2004 (20). Eleven and 14 sites were tested in 2006 and 2008, respectively (21, 22). From 2010 to 2018, ASD monitoring for children aged 8 years was available at 11 states (11, 23–26). The ADDM Network reports provide data on region-, sex-, and age-based differences in ASD prevalence and diagnostic challenges involved, including best practice guidance. In addition, the ADDM Network updates biennially ASD prevalence data in children aged 4 years, providing foundational public health evidence. In a study published in 2017, Soke et al. investigated early detection rate of ASD in infants born between 2002 and 2006 and examined at five ADDM Network sites, showing differences in detection standards among regions (27). Yingling et al. reported in 2019 that more than half of the United States' counties lack a Board Certified Behavior Analyst (BCBA) qualified to provide behavioral and psychological assessments and interventions due to differences in reimbursement ratios (28). This finding suggests that many children with ASD do not receive the required support.

The research on the prevalence monitoring of autism is still worthy of attention. Disability monitoring networks are an essential source of research data and clinical guidance, informing research and treatment strategies, and helping improve patient outcomes. This relies on scientific diagnostic tools.

#### Caregivers

Research into caregivers of children with ASD covers aspects such as caregiver demographic characteristics and views on treatment, nursing training, and health problem, quality of life, and family cohesion surveys, as well as input into specialist nursing and intelligent care system development.

Demographic characteristics of patients, caregivers, and communities affect the perception of patients with ASD. The mothers of 396 children from Singapore completed a quantitative checklist for autism in adults, revealing that child sex, cognitive function, birth order, mother age, and ethnicity were not characteristics of autism reported by caregivers. Delayed or impaired language development in the child and depressive symptoms and low education level in the mother were associated with social autism (29). Caregivers able to predict child problematic behavior may take action to minimize this risk. However, such predictions are challenging for caregivers that do not receive BCBA support, in which case the use of artificial intelligence technologies may be useful. Zheng et al. proposed a machine learning-based framework that helps predict problem behavior (30). Following an early diagnosis, caregivers of children with ASD need specialist education to help meet child needs. This approach also encourages social interaction among caregivers (31), which helps improve caregiver physical and psychological health and quality of life (32, 33). In 2021, Nie et al. proposed an immersive interaction system based on augmented reality to help autistic children practice social attention skills (34).

### Limitations

There are some limitations to this study. First, we downloaded only citations from the WoSCC database. The findings may differ if citations from other databases are included. Second, despite efforts to reduce bias in this study, no study method can reduce bias present in the original studies. Third, this study only included research findings published between 2012 and 2021. Some relevant studies are on-going, precluding inclusion in the present study, which may subject these results to the effects of time lag. Some of the most recent research hotspots require manual reading to be discovered. The use of artificial intelligence in medicine is increasing, providing novel opportunities to codify existing bias and change practice (35); the use of these technologies in ASD research and care is particularly promising (36).

## Conclusions

Disability monitoring networks and caregiver-focused questions are the leading areas of research in childhood ASD, aiming to improve diagnosis, interventions, and outcomes. Many questions in this field remain unanswered. Eleven sites across the United States provide ASD prevalence data, which may support policy development and clinical practice. These data may help establish rehabilitation intervention systems for caregivers, supporting disease management. The use of artificial intelligence-based technologies may support research and clinical practice, including studies aimed at elucidating neurobiomarkers of ASD. Remote assessment and intervention guidance is required in the time of pandemics (37, 38).

## Data availability statement

The original contributions presented in the study are included in the article/supplementary files, further inquiries can be directed to the corresponding author.

## Author contributions

JZ, YL, and FZ acquired, analyzed the data, and drafted the manuscript. FF, XD, and XingW analyzed the data. MW and XiaoW designed the research, acquired the article information, and revised the manuscript. All authors agreed to be accountable for the content of the work. All authors contributed to the article and approved the submitted version.

## Funding

The work was financially supported the Key R&D and Promotion Projects in Henan Province (222102310615) and Henan Province Medical Science and Technology Key Project Jointly Constructed by Province and Ministry (SBGJ202102189).

## Conflict of interest

The authors declare that the research was conducted in the absence of any commercial or financial relationships that could be construed as a potential conflict of interest.

## Publisher's note

All claims expressed in this article are solely those of the authors and do not necessarily represent those of their affiliated organizations, or those of the publisher, the editors and the reviewers. Any product that may be evaluated in this article, or claim that may be made by its manufacturer, is not guaranteed or endorsed by the publisher.

## References

[1] Katz L Nayar K Garagozzo A Schieszler-Ockrassa C Paxton J. Changes in autism nosology: the social impact of the removal of asperger's disorder from the diagnostic and statistical manual for mental disorders, fifth edition (DSM-5). J. Autism Dev. Disord. (2020) 50:3358–66. 10.1007/s10803-019-04233-4 31535343

[2] World Health Organization. Autism Spectrum Disorders. WHO (2020).

[3] Cai RY Richdale AL Dissanayake C Uljarevic M. Resting heart rate variability, emotion regulation, psychological wellbeing and autism symptomatology in adults with and without autism. Int J Psychophysiol. (2019) 137:54–62. 10.1016/j.ijpsycho.2018.12.010 30578793

[4] Leader G O'Reilly M Gilroy SP Chen JL Ferrari C Mannion A. Comorbid feeding and gastrointestinal symptoms, challenging behavior, sensory issues, adaptive functioning and quality of life in children and adolescents with autism spectrum disorder. Dev Neurorehabil. (2021) 24:35–44. 10.1080/17518423.2020.1770354 32496834

[5] Kanner L. Autistic disturbances of affective contact. Nervous Child. (1943) 2:217–50. 4880460

[6] Rutter M. Diagnosis and definition of childhood autism. J Autism Childhood Schizophrenia. (1978) 8:139–61. 10.1007/BF01537863 670129

[7] Pichot P. [DSM-III: the 3d edition of the Diagnostic and Statistical Manual of Mental Disorders from the American Psychiatric Association]. Rev Neurol. (1986) 142:489–99. 3787052

[8] Bell CC. DSM-IV: diagnostic and statistical manual of mental disorders. JAMA. (1994) 272:828–9. 10.1001/jama.1994.0352010009604625996397

[9] Lai MC Lombardo MV Baron-Cohen S. Autism. Lancet. (2014) 383:896–910. 10.1016/S0140-6736(13)61539-1 24074734

[10] American Psychiatric Association. DC. DSM-5 Task Force Diagnostic and Statistical Manual of Mental Disorders: DSM-5. Washington, DC: American Psychiatric Association (2013).

[11] Baio J Wiggins L Christensen DL Maenner MJ Daniels J Warren Z. Prevalence of autism spectrum disorder among children aged 8 years - autism and developmental disabilities monitoring network, 11 sites, United States, 2014. MMWR Surveill Summ. (2018) 67:1–23. 10.15585/mmwr.ss6706a1 29701730 PMC5919599

[12] Hayes SA Watson SL. The impact of parenting stress: a meta-analysis of studies comparing the experience of parenting stress in parents of children with and without autism spectrum disorder. J Autism Dev Disord. (2013) 43:629–42. 10.1007/s10803-012-1604-y 22790429

[13] Dawson G Rogers S Munson J Smith M Winter J Greenson J. Randomized, controlled trial of an intervention for toddlers with autism: the early start denver model. Pediatrics. (2010) 125:e17–23. 10.1542/peds.2009-0958 19948568 PMC4951085

[14] Elsabbagh M Divan G Koh YJ Kim YS Kauchali S Marcin C. Global prevalence of autism and other pervasive developmental disorders. Autism Res. (2012) 5:160–79. 10.1002/aur.239 22495912 PMC3763210

[15] Wong C Odom SL Hume KA Cox AW Fettig A Kucharczyk S. Evidence-based practices for children, youth, and young adults with autism spectrum disorder: a comprehensive review. J Autism Dev Disord. (2015) 45:1951–66. 10.1007/s10803-014-2351-z 25578338

[16] Schreibman L Dawson G Stahmer AC Landa R Rogers SJ McGee GG. Naturalistic developmental behavioral interventions: empirically validated treatments for autism spectrum disorder. J Autism Dev Disord. (2015) 45:2411–28. 10.1007/s10803-015-2407-8 25737021 PMC4513196

[17] Loomes R Hull L Mandy WPL. What is the male-to-female ratio in autism spectrum disorder? A systematic review and meta-analysis. J Am Acad Child Adolesc Psychiatry. (2017) 56:466–74. 10.1016/j.jaac.2017.03.013 28545751

[18] Christensen DL Bilder DA Zahorodny W Pettygrove S Durkin MS Fitzgerald RT. Prevalence and characteristics of autism spectrum disorder among 4-year-old children in the autism and developmental disabilities monitoring network. J Dev Behav Pediatrics. (2016) 37:1–8. 10.1097/DBP.0000000000000235 26651088

[19] Control Centers for Disease, Prevention. (2007). Prevalence of autism spectrum disorders–autism and developmental disabilities monitoring network, six sites, United States, 2000. MMWR Surveill Summ. (2007) 56:1–11. Available online at: https://www.cdc.gov/mmwr/preview/mmwrhtml/ss5601a1.htm 17287714

[20] Centers for Disease Control and Prevention. Prevalence of autism spectrum disorders–autism and developmental disabilities monitoring network, 14 sites, United States. MMWR Surveill Summ. (2007) 56:12–28. Available online at: https://www.cdc.gov/mmwr/preview/mmwrhtml/ss5601a2.htm 17287715

[21] Control Centers for Disease and Prevention. Prevalence of autism spectrum disorders-autism and developmental disabilities monitoring network, United States. MMWR Surveill Summ. (2009) 58:1–20. Available online at: https://www.cdc.gov/mmwr/preview/mmwrhtml/ss5810a1.htm 20023608

[22] Autism, Developmental Disabilities Monitoring Network Surveillance Year Principal, Investigators. Prevalence of autism spectrum disorders—autism and developmental disabilities monitoring network, 14 sites, United States. MMWR Surveill Summ. (2012) 61:1–19. Available online at: https://www.cdc.gov/mmwr/preview/mmwrhtml/ss6103a1.htm 22456193

[23] Baio J. Prevalence of autism spectrum disorder among children aged 8 years-autism and developmental disabilities monitoring network, 11 sites, United States 2010. MMWR Surveill Summ. (2014) 63:1–21. Available online at: https://www.cdc.gov/mmwr/preview/mmwrhtml/ss6302a1.htm 24670961

[24] Christensen DL Baio J Braun KV Bilder D Charles J Constantino JN. Prevalence and characteristics of autism spectrum disorder among children aged 8 years - autism and developmental disabilities monitoring network, 11 sites, united states, 2012. MMWR Surveill Summ. (2016) 65:1–23. 10.15585/mmwr.ss6503a1 27031587 PMC7909709

[25] Maenner MJ Shaw KA Bak J Washington A Patrick M DiRienzo M. Prevalence of autism spectrum disorder among children aged 8 years - autism and developmental disabilities monitoring network, 11 sites, United States, 2016. MMWR Surveill Summ. (2020) 69:1. 10.15585/mmwr.ss6904a1 32214087 PMC7119644

[26] Maenner MJ Shaw KA Bakian AV Bilder DA Durkin MS Esler A. Prevalence and characteristics of autism spectrum disorder among children aged 8 years - autism and developmental disabilities monitoring network, 11 sites, United States, 2018. MMWR Surveill Summ. (2021) 70:16. 10.15585/mmwr.ss7011a1 34855725 PMC8639024

[27] Soke GN Maenner MJ Christensen D Kurzius-Spencer M Schieve LA. Brief report: estimated prevalence of a community diagnosis of autism spectrum disorder by age 4 years in children from selected areas in the United States in 2010: evaluation of birth cohort effects. J Autism Dev Disord. (2017) 47:1917–22. 10.1007/s10803-017-3094-4 28342162 PMC5650225

[28] Yingling ME Ruther MH Dubuque EM David SM. County-level variation in geographic access to board certified behavior analysts among children with autism spectrum disorder in the United States. Autism. (2021) 25:1734–45. 10.1177/13623613211002051 33740869

[29] Goh DA Gan D Kung J Baron-Cohen S Allison C Chen H. Child, maternal and demographic factors influencing caregiver-reported autistic trait symptomatology in toddlers. J Autism Dev Disord. (2018) 48:1325–37. 10.1007/s10803-018-3471-7 29388148

[30] Zheng ZBK Staubitz JE Weitlauf AS Staubitz J Pollack M Shibley L. A Predictive Multimodal Framework to Alert Caregivers of Problem Behaviors for Children with ASD (PreMAC). Sensors. (2021) 21:19. 10.3390/s21020370 33430371 PMC7826816

[31] Recio P Molero F Garcia-Ael C Perez-Garin D. Perceived discrimination and self-esteem among family caregivers of children with autism spectrum disorders (ASD) and children with intellectual disabilities (ID) in Spain: the mediational role of affiliate stigma and social support. Res Dev Disabil. (2020) 105:8. 10.1016/j.ridd.2020.103737 32679389

[32] Chen JY Strodl E Huang LH Chen YJ Yang GY Chen WQ. Early electronic screen exposure and autistic-like behaviors among preschoolers: the mediating role of caregiver-child interaction, sleep duration and outdoor activities. Children. (2020) 7:15. 10.3390/children7110200 33126543 PMC7692375

[33] DesChamps TD Ibanez LV Edmunds SR Dick CC Stone WL. Parenting stress in caregivers of young children with ASD concerns prior to a formal diagnosis. Autism Res. (2020) 13:82–92. 10.1002/aur.2213 31593362

[34] Nie GT Ullal A Zheng Z Swanson AR Weitlauf AS Warren ZE. An immersive computer-mediated caregiver-child interaction system for young children with autism spectrum disorder. IEEE Trans Neural Syst Rehabil Eng. (2021) 29:884–93. 10.1109/TNSRE.2021.3077480 33945481 PMC8189254

[35] Straw I. The automation of bias in medical Artificial Intelligence (AI): decoding the past to create a better future. Artif Intell Med. (2020) 110:3. 10.1016/j.artmed.2020.101965 33250145

[36] Mahmood A Nayak P English C Deshmukh A Manikandan NSU Solomon JM. Adherence to home exercises and rehabilitation (ADHERE) after stroke in low-to-middle-income countries: a randomized controlled trial. Top Stroke Rehabil. (2021) 29:1–11. 10.1080/10749357.2021.1940800 34180370

[37] Newbutt N Schmidt MM Riva G Schmidt C. The possibility and importance of immersive technologies during COVID-19 for autistic people. J Enabl Technol. (2020) 14:187–99. 10.1108/JET-07-2020-0028

[38] Jan RK Rihs TA Kojovic N Sperdin HF Franchini M Custo A. Neural processing of dynamic animated social interactions in young children with autism spectrum disorder: a high-density electroencephalography study. Front Psychiatry. (2019) 10:13. 10.3389/fpsyt.2019.00582 31507462 PMC6714589

