# Erratum: Bibliometric analysis of research themes and trends in childhood autism spectrum disorders from 2012 to 2021

**DOI:** 10.3389/fpubh.2022.1058389

**Published:** 2022-11-28

**Authors:** Frontiers Production Office

**Affiliations:** Frontiers Media SA, Lausanne, Switzerland

**Keywords:** autism spectrum disorders, children, bibliometrics, CiteSpace, VOSviewer

Due to a production error, there was an error in the affiliation of Fangqin Fei. Instead of “Department of Nursing, Huzhou Maternal and Child Health Care Hospital, Huzhou, China,” it should be “Department of Nursing, The First Affiliated Hospital of Huzhou University, Huzhou, China.”

The publisher apologizes for this mistake. The original version of this article has been updated.

